# Simultaneous multi slice (SMS) balanced steady state free precession first-pass myocardial perfusion cardiovascular magnetic resonance with iterative reconstruction at 1.5 T

**DOI:** 10.1186/s12968-018-0502-7

**Published:** 2018-12-10

**Authors:** Muhummad Sohaib Nazir, Radhouene Neji, Peter Speier, Fiona Reid, Daniel Stäb, Michaela Schmidt, Christoph Forman, Reza Razavi, Sven Plein, Tevfik F. Ismail, Amedeo Chiribiri, Sébastien Roujol

**Affiliations:** 1 School of Biomedical Engineering and Imaging Sciences, King’s College London, 3rd Floor Lambeth Wing, St Thomas’ Hospital, Westminster Bridge Road, London, SW1 7EH UK; 2 MR Research Collaborations, Siemens Healthcare Limited, Frimley, UK; 3 Siemens Healthcare, Erlangen, Germany; 4 Division of Health and Social Care Research, King’s College London, London, UK; 5 Siemens Healthcare Pty Ltd, Melbourne, Australia; 6 Leeds Institute of Cardiovascular and Metabolic Medicine, LIGHT Laboratories, Clarendon Way, University of Leeds, Leeds, LS2 9JT UK

**Keywords:** Cardiovascular magnetic resonance, Myocardial perfusion imaging, Simultaneous multi-slice, Image acceleration, Iterative reconstruction

## Abstract

**Background:**

Simultaneous-Multi-Slice (SMS) perfusion imaging has the potential to acquire multiple slices, increasing myocardial coverage without sacrificing in-plane spatial resolution. To maximise signal-to-noise ratio (SNR), SMS can be combined with a balanced steady state free precession (bSSFP) readout. Furthermore, application of gradient-controlled local Larmor adjustment (GC-LOLA) can ensure robustness against off-resonance artifacts and SNR loss can be mitigated by applying iterative reconstruction with spatial and temporal regularisation. The objective of this study was to compare cardiovascular magnetic resonance (CMR) myocardial perfusion imaging using SMS bSSFP imaging with GC-LOLA and iterative reconstruction to 3 slice bSSFP.

**Methods:**

Two contrast-enhanced rest perfusion sequences were acquired in random order in 8 patients: 6-slice SMS bSSFP and 3 slice bSSFP. All images were reconstructed with TGRAPPA. SMS images were also reconstructed using a non-linear iterative reconstruction with L1 regularisation in wavelet space (SMS-iter) with 7 different combinations for spatial (λ_σ_) and temporal (λ_τ_) regularisation parameters. Qualitative ratings of overall image quality (0 = poor image quality, 1 = major artifact, 2 = minor artifact, 3 = excellent), perceived SNR (0 = poor SNR, 1 = major noise, 2 = minor noise, 3 = high SNR), frequency of sequence related artifacts and patient related artifacts were undertaken. Quantitative analysis of contrast ratio (CR) and percentage of dark rim artifact (DRA) was performed.

**Results:**

Among all SMS-iter reconstructions, SMS-iter 6 (λ_σ_ 0.001 λ_τ_ 0.005) was identified as the optimal reconstruction with the highest overall image quality, least sequence related artifact and higher perceived SNR. SMS-iter 6 had superior overall image quality (2.50 ± 0.53 vs 1.50 ± 0.53, *p* = 0.005) and perceived SNR (2.25 ± 0.46 vs 0.75 ± 0.46, *p* = 0.010) compared to 3 slice bSSFP. There were no significant differences in sequence related artifact, CR (3.62 ± 0.39 vs 3.66 ± 0.65, *p* = 0.88) or percentage of DRA (5.25 ± 6.56 vs 4.25 ± 4.30, *p* = 0.64) with SMS-iter 6 compared to 3 slice bSSFP.

**Conclusions:**

SMS bSSFP with GC-LOLA and iterative reconstruction improved image quality compared to a 3 slice bSSFP with doubled spatial coverage and preserved in-plane spatial resolution. Future evaluation in patients with coronary artery disease is warranted.

**Electronic supplementary material:**

The online version of this article (10.1186/s12968-018-0502-7) contains supplementary material, which is available to authorized users.

## Background

First-pass contrast enhanced myocardial perfusion cardiovascular magnetic resonance (CMR) is recommended in international guidelines for ischaemia testing in patients with intermediate risk of coronary artery disease (CAD) [1, 2]. A recent meta-analysis demonstrated a sensitivity of 89% and specificity of 76% for the detection of angiographically defined CAD [3].

Various pulse sequences are used in clinical practice and guidelines recommend the acquisition of at least 3 short axis slices with an in-plane resolution of <3x3mm [4]. The sequences used typically employ electrocardiogram (ECG) triggering, saturation pre-pulses, and three to four sequentially acquired 2D slices distributed over a single heartbeat. Alternatively, 3D techniques have been proposed to achieve whole heart coverage [5, 6] but are usually associated with reduced in-plane spatial resolution, longer imaging readout and are more susceptible to respiratory motion [6]. There is considerable debate as to whether in-plane spatial resolution or spatial coverage are more important for clinical practice [5].

Simultaneous multi-slice (SMS) imaging is an alternative data acquisition strategy [7–11] with potential to increase spatial coverage without sacrificing in-plane spatial resolution. Using multiband radiofrequency (RF) pulses, separate anatomical slices are excited simultaneously. By means of Controlled Aliasing in Parallel Imaging Results in Higher Acceleration (CAIPIRINHA) [11], the simultaneously excited slices are shifted with respect to each other in image space, which facilitates their separation using parallel imaging techniques [12–14]. Hence, multiple slice acquisitions can be performed in the same duration as a single slice acquisition.

SMS balanced steady state free precession (bSSFP) can be achieved using linear slice specific RF phase cycles with different RF phase increments between succeeding RF pulses in the simultaneously excited slices [15]. Using different phase cycles in the individual slices renders the frequency response slice specific and results in an increased sensitivity to field inhomogeneities. Gradient controlled local Larmor adjustment (GC-LOLA) can be used to restore the frequency response and thus mitigate the effects of field inhomogeneities by unbalancing the gradients along the slice select direction [16].

Standard parallel imaging techniques such as with GRAPPA [12] and SENSE [13] are associated with signal-to-noise (SNR) degradation in the presence of noise and high undersampling factors. The use of prior information in the form of additional regularisation constraints in the reconstruction can be used to improve the quality and SNR of the reconstructed images [17, 18]. Regularisation can be achieved by assuming the sparsity of the data in a given transform domain [19, 20], as developed in the compressed sensing theory [21]. The reconstruction problem is in this case often formulated as an inverse problem which can be solved using an iterative reconstruction process.

The objective of this study was to determine the feasibility of first-pass myocardial perfusion CMR using SMS with a bSSFP sequence, GC-LOLA and iterative reconstruction and compare to a 3 slice bSSFP sequence in patients.

## Methods

### Study population

Patients (*n* = 8) referred for a clinically indicated contrast enhanced non-stress CMR scan were prospectively recruited to undergo two additional rest myocardial perfusion scans. The clinical indication for the CMR was for assessment of possible cardiomyopathy (*n* = 5) and assessment of left ventricular (LV) volumes and function (*n* = 3). Exclusion criteria were contraindication to gadolinium contrast agent or CMR (non CMR safe metallic implant). The study was approved by the National Research Ethics Service (15/NS/0030) with written informed consent obtained from all patients for inclusion in the study and additional imaging during their clinical CMR scan.

### Perfusion protocol

Prior to imaging, patients were coached for breath holding and instructed to breath hold during first-pass of contrast. Two rest perfusions scans were acquired in each patient for 3 slice bSSFP and 6-slice SMS, separated by a minimum of 15 min to allow for contrast washout. The sequence order was alternated in successive patients in order to negate the effect of higher baseline signal following contrast administration of the first perfusion sequence.

Contrast was administered using a dual bolus technique as previously described [22], with 0.0075 + 0.075 mmol/kg of body weight gadolinium (gadobutrol, Gadovist, Bayer Healthcare, Berlin, Germany). The prebolus and main bolus contrast were separated by a 25 s delay and injected at a rate of 4 mL/s followed by a 25 ml flush of normal saline. Each injection was performed by a power injector (Spectris Solaris® EP, Medrad, Inc., Warrendale, Pennsylvania, USA).

Slice locations were planned using the systolic phase of the 4 and 2 chamber cine images, and the 3 chamber cine image to ensure the basal slice did not encroach on the LV outflow tract (LVOT). For the 3 slice bSSFP approach, the ‘3 of 5’ rule was used in order to establish basal, mid and apical slices [23]. This involves planning of 5 equidistant slices from proximal basal LV from the mitral valve annulus to outer boundary of the LV apex in systole, after which the number of slices is adjusted to 3. For the SMS approach, a ‘6 of 10’ rule was employed to obtain 6 slice locations from the basal LV to the apex. This involved planning 10 equidistant slices from the basal LV to apex in the 4, 2 and 3 chamber cine view in systole and then switching to 6 slices in that orientation.

### Data acquisition and image reconstruction

Imaging was undertaken at 1.5 T (Magnetom Aera, Siemens Healthineers, Erlangen, Germany). Sequence parameters were matched between the two sequences: field-of-view (FOV) 332 × 332 mm, acquired voxel size 1.9 × 1.9 mm, slice thickness 10 mm, flip angle 50°, bandwidth 1093 Hz/px, in-plane acceleration 2.5, inversion time (TI) 95 ms [bSSFP] 130 ms [SMS], repetition time (TR) 2.5 ms [bSSFP] 2.9 ms [SMS], echo time (TE) 1.04 ms [bSSFP] 1.24 ms [SMS]. Standard bSSFP imaging was acquired with 3 short axis slices. SMS images were acquired with a prototype of a SMS-bSSFP sequence that implements the GC-LOLA technique with a slice acceleration factor of 2 to acquire 6 short axis slices per heartbeat. Two different RF phase cycles were used for the SMS sequences using two different phase increments of -π/2 for slice 1 (i.e. 0, 3π/2, π, π/2, 0, …) and π/2 Slice 2 (i.e. 0, π/2, π, 3π/2, 0, …). GC-LOLA was used to compensate for the slice specific shifts of the bSSFP frequency response induced by these RF phase cycles as previously described [16].

SMS data were reconstructed using a prototype of a non-linear iterative reconstruction with L1 regularisation in wavelet space (referred to as SMS-iter) [19, 21, 24], implemented inline in the standard reconstruction framework of the scanner. Spatial and temporal L1 regularisation was performed for the frames {**x**_*t*_}_*t* = 1, …, *T*_ for all time points *T* as similarly in previous work [25]:

1 $$ {\left\{{\mathbf{x}}_t\right\}}_{t=1,\dots, T}={\mathrm{argmin}}_{\left\{{\mathbf{x}}_t\right\}}{\sum}_{t=1}^T\left({\left\Vert {\mathbf{A}}_t{\mathbf{x}}_t-{\mathbf{y}}_t\left\Vert {}_2^2\right.+{\lambda}_{\sigma}\left\Vert {\mathbf{W}}_{\sigma }{\mathbf{x}}_t\right.\right\Vert}_1\right)+{\lambda}_{\tau }{\left\Vert {\mathbf{W}}_{\tau }{\left\{{\mathbf{x}}_1^{\top },\dots, {\mathbf{x}}_T^{\top}\right\}}^{\top}\right\Vert}_1, $$

**A**_*t*_is the system matrix for time *t* consisting of the corresponding sampling pattern, Fourier transform, and coil sensitivity maps for the local receiver coil elements. The measured data for time *t* is denoted by **y**_*t*_, *λ*_*σ*_ and *λ*_*τ*_ are the spatial and temporal regularisation parameters respectively. **W**_*σ*_ and **W**_*τ*_ are the corresponding spatial and temporal Wavelet transforms respectively. Equation 1 is solved using Fast Iterative Shrinkage Thresholding Algorithm (FISTA) optimisation [26] alternating a gradient descent step for the quadratic terms and the evaluation of the proximal operator of the L1 terms. The proximal step was computed using the memory-efficient Chambolle-Pock algorithm [27], and a total of 40 iterations were used for each reconstruction.

To evaluate and optimise the weight of the spatio-temporal regularisation terms (λ_σ_ and λ_τ_), seven different reconstructions were performed for each patient. The first four reconstructions evaluated the impact of increasing both spatial and temporal regularisation as follows with an approximate doubling of the regularisation factors in succession: SMS-iter 1 (λ_σ_ 0.0005 λ_τ_ 0.0005), SMS-iter 2 (λ_σ_ 0.001 λ_τ_ 0.001), SMS-iter 3 (λ_σ_ 0.0025 λ_τ_ 0.0025), SMS-iter 4 (λ_σ_ 0.005 λ_τ_ 0.005). The subsequent three reconstructions evaluated the impact of using a greater weighting for temporal regularisation. As SMS-iter 2 was found superior among the first four reconstructions (as described in the results section), the spatial regularisation factor (λ_σ_) was kept constant to 0.001, whilst the temporal regularisation factor (λ_τ_) was almost doubled in succession: SMS-iter 5 (λ_σ_ 0.001 λ_τ_ 0.002), SMS-iter 6 (λ_σ_ 0.001 λ_τ_ 0.005), SMS-iter 7 (λ_σ_ 0.001 λ_τ_ 0.01). For comparison, 3 slice bSSFP and SMS data were reconstructed using standard TGRAPPA reconstruction [28].

### Qualitative image assessment

Qualitative image analysis was undertaken in consensus by two experienced CMR readers (AC and TI) with more than 10 years’ experience in CMR each using a standardised rating scale (Table 1). Overall diagnostic image quality, perceived SNR, ‘sequence related’ artifact and ‘patient related’ artifact were ranked for each perfusion dataset (Table 1). The CMR readers were blinded to the clinical details of the patients and to the method of reconstruction for SMS imaging. Images were presented to readers in randomised order.

**Table 1:** Four categories for qualitative image quality assessment. (A) overall diagnostic image quality (range 0–3), (B) sequence-related artifact (7 criteria range 0–3, maximum total score 21), (C) patient related artifact (2 criteria with range 0–3, total maximum score 6) and (D) perceived signal-to-noise ratio (SNR) (range 0–3). (B) Score for sequence related artifact relates to total number of slices acquired (artifact present in 1, 2 or 3 slices would score 1, 2 and 3 respectively for 3 slice balanced steady state free precession (bSSFP); artifact present in 1–2, 3–4 or 5–6 slices would score 1, 2 and 3 respectively for 6-slice SMS)

Qualitative Criteria for perfusion Imaging					
	0	1	2	3	maximum score
A. Overall Diagnostic Image Quality					
	poor image quality and non-diagnostic	major artifact present but not limiting diagnosis	minor artifact present but not limiting diagnosis	excellent	3
					3
B. Sequence related artifact					
Wrap around	none	1 slice	2 slices	3 slices	3
Respiratory ghosting	none	1 slice	2 slices	3 slices	3
Cardiac ghosting	none	1 slice	2 slices	3 slices	3
Image blurring	none	1 slice	2 slices	3 slices	3
Metallic artifact	none	1 slice	2 slices	3 slices	3
Banding artifact	none	1 slice	2 slices	3 slices	3
Cardiac Motion	none	1 slice	2 slices	3 slices	3
					21
C. Patient / equipment related artifact					
Breathing motion	none	minor artifact present but not limiting diagnosis	major artifact present but not limiting diagnosis	non-diagnostic	3
ECG mistriggers	none	1 mistriggers	2 mistriggers	> 2 mistriggers	3
					6
D. Perceived Signal-to-Noise Ratio (SNR)					
	very poor SNR non-diagnostic image quality	minor noise present but not limiting diagnosis	major noise present but not limiting diagnosis	high SNR with excellent image quality	3
					3

### Quantitative assessment

In order to provide quantitative metrics for image quality, contrast ratio (CR) and extent of dark rim artifact (DRA) were evaluated. For CR, regions of interest (ROI) from the mid ventricular slice of the perfusion sequence were taken, in order to avoid partial volume effects of sampling at the basal or apical slice. ROIs for the myocardium were obtained with manual contouring of endocardium and epicardium and of the LV blood pool with careful exclusion of papillary muscles. CR was calculated as the ratio of peak LV blood pool SI: peak myocardial SI per slice. The extent of DRA was defined as percentage of the circumferential DRA of the total endocardium.

### Statistical analysis

All statistical analyses were performed using SPSS Statistics 23 (International Business Machines Inc., Armonk, New York, USA). Results are expressed as mean ± standard deviation unless otherwise specified. Qualitative image quality, sequence related artifact and patient related artifact scores, and perceived SNR were compared between methods using the Wilcoxon signed ranks test for paired ordinal data. Mean CR scores were compared between methods using paired t tests, having checked the assumption of normally distributed differences. All statistical tests were two-tailed and *p*-values < 0.05 were considered significant.

## Results

### Study population

All patient scans were completed successfully. The CMR examination was normal in 6 of the patients. Two patients were found to have sustained previous myocardial infarction and had impaired LV systolic function. Participant characteristics are shown in Table 2. Two patients had suboptimal breath holds during contrast administration, reflecting real-world clinical practice.

**Table 2:** Study participant characteristics for 8 patients. Results are mean ± standard deviation or number (%), as specified

Age (years)	50 ± 22
Male: number (%)	6 (75%)
Body Mass Index (kg/m^2^)	25 ± 5
Previous MI: number (%)	2 (25%)
LVEF (%)	52 ± 16
Indexed LV EDV (ml/m^2^)	95 ± 27
RVEF (%)	57 ± 10
Indexed RV EDV (ml/m^2^)	80 ± 23
Scar present: number (%)	2 (25%)
Resting Heart Rate (beats/min)	65 ± 14

*EDV* end-diastolic volume; *EF* ejection fraction; *LV* left ventricular; *MI* myocardial infarction; *RV* right ventricular

### Slice location and image reconstruction

Using the ‘3 of 5’ approach for slice location, reliable basal, mid and apical slices were generated for all 3 slice bSSFP images as defined by established criteria for slice location [29]. The ‘6 of 10’ approach generated reliable basal, mid and apical slices in 46/48 (96%) of all SMS slices. Of the remaining 2/48 slices, in two patients, the basal LV slice included part of the LVOT. All 3 slice bSSFP data were reconstructed with TGRAPPA and SMS data were successfully reconstructed with TGRAPPA and the different iterative reconstruction parameters on the scanner platform. The iterative reconstruction of the SMS images took approximately 10 min on the scanner console.

### Optimum SMS iterative reconstruction

As the weighting of spatial (λ_σ_) and temporal (λ_τ_) regularisation were both sequentially increased (SMS-iter 1–4, see Table 3), there was a trend towards increased perceived SNR and CR. However, for high spatial and temporal regularisation (SMS-iter 3 and 4), higher sequence related artifact (due to increased frequency of respiratory ghosting and image blurring) was observed resulting in a reduction in overall image quality. Among these four SMS iterative reconstructions, SMS-iter 2 was used as a basis for further investigation of temporal regularisation.

**Table 3:** Image quality assessment of images produced from iterative reconstruction of SMS images with different parameters, for 8 patients (see Table 1 for definition of rating scales). λ_σ_ indicates the degree of spatial regularisation, whilst λ_τ_ indicates the extent of temporal regularisation. SMS-iter 6 (λ_σ_ 0.001 λ_τ_ 0.005) had the highest overall image quality and the lowest amount of sequence related artifact. Results are mean ± standard deviation. Contrast ratio calculated as the ratio peak blood pool signal intensity: peak myocardial signal intensity

Iterative reconstruction parameters	Overall Image Quality	Perceived SNR	Sequence Related Artifact	Patient Related Artifact	Contrast Ratio
SMS-iter 1 (λ_σ_ 0.0005 λ_τ_ 0.0005)	1.63 ± 0.74	1.63 ± 0.52	0.69 ± 1.10	0.89 ± 1.36	3.64 ± 0.37
SMS-iter 2 (λ_σ_ 0.001 λ_τ_ 0.001)	1.88 ± 0.35	1.63 ± 0.52	0.31 ± 0.59	0.89 ± 1.36	3.73 ± 0.44
SMS-iter 3 (λ_σ_ 0.0025 λ_τ_ 0.0025)	1.50 ± 0.53	2.00 ± 0.00	0.63 ± 1.27	1.00 ± 1.41	3.80 ± 0.47
SMS-iter 4 (λ_σ_ 0.005 λ_τ_ 0.005)	1.25 ± 0.46	1.88 ± 0.35	1.88 ± 1.62	1.00 ± 1.41	3.93 ± 0.51
SMS-iter 5 (λ_σ_ 0.001 λ_τ_ 0.0025)	2.13 ± 0.35	2.13 ± 0.35	0.25 ± 0.38	0.78 ± 1.40	3.63 ± 0.40
SMS-iter 6 (λ_σ_ 0.001 λ_τ_ 0.005)	2.50 ± 0.53	2.25 ± 0.46	0.13 ± 0.35	0.56 ± 1.01	3.62 ± 0.39
SMS-iter 7 (λ_σ_ 0.001 λ_τ_ 0.01)	2.00 ± 0.53	2.25 ± 0.46	0.56 ± 0.86	0.67 ± 1.32	3.64 ± 0.40

**Fig. 1:**
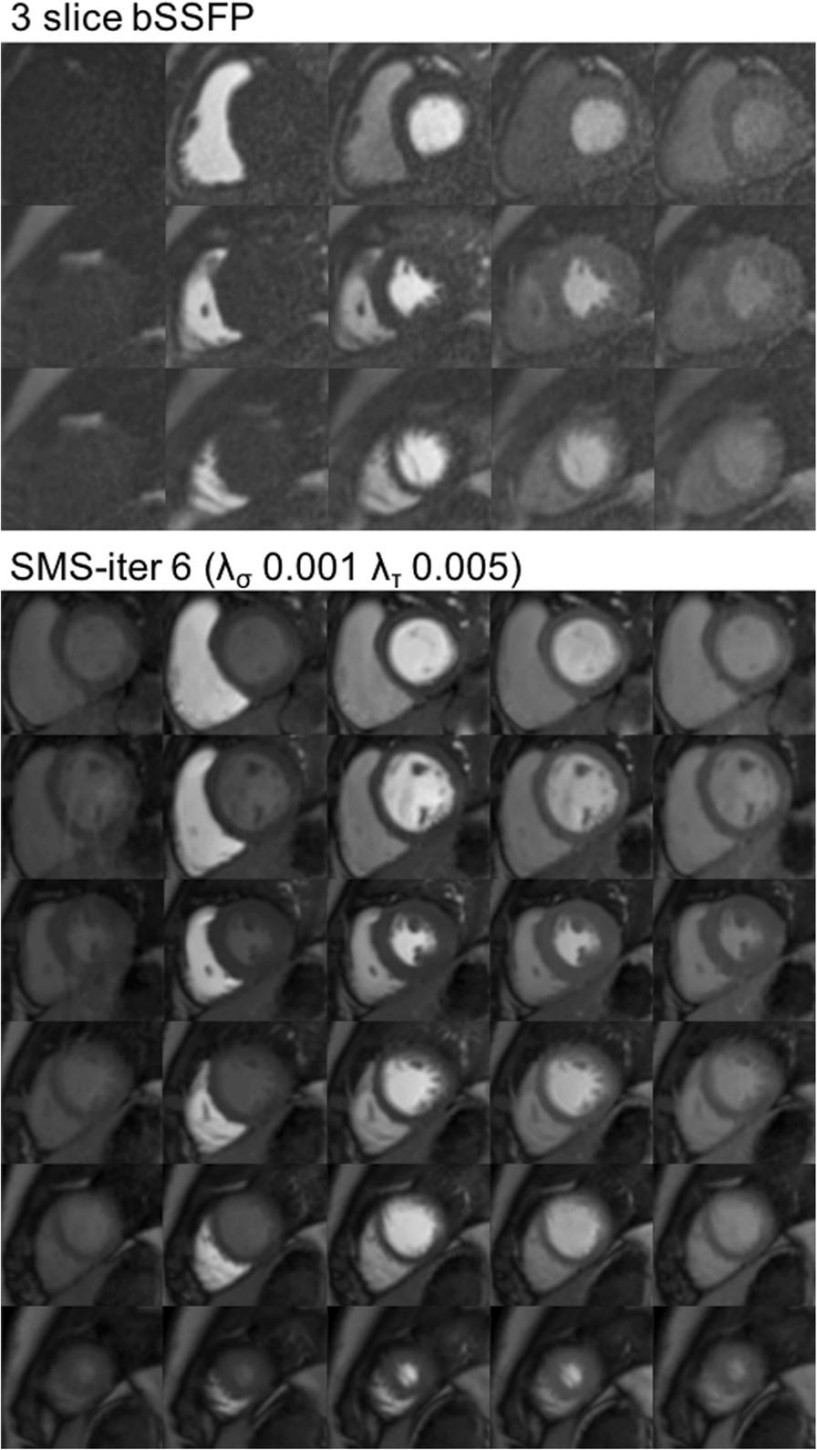
Dynamic perfusion series following contrast administration using 3 slice balanced steady state free precession (bSSFP) and simultaneous multi-slice (SMS)-iter 6 (λ_σ_ 0.001 λ_τ_ 0.005) in patient 1. Top to bottom: base to apex. Left to right: baseline images, contrast transit through right ventricle (RV), left ventricular (LV) blood pool, peak myocardial and washout. SMS-iter 6 (λ_σ_ 0.001 λ_τ_ 0.005) had better subjective image quality compared to 3 slice bSSFP

**Fig. 2:**
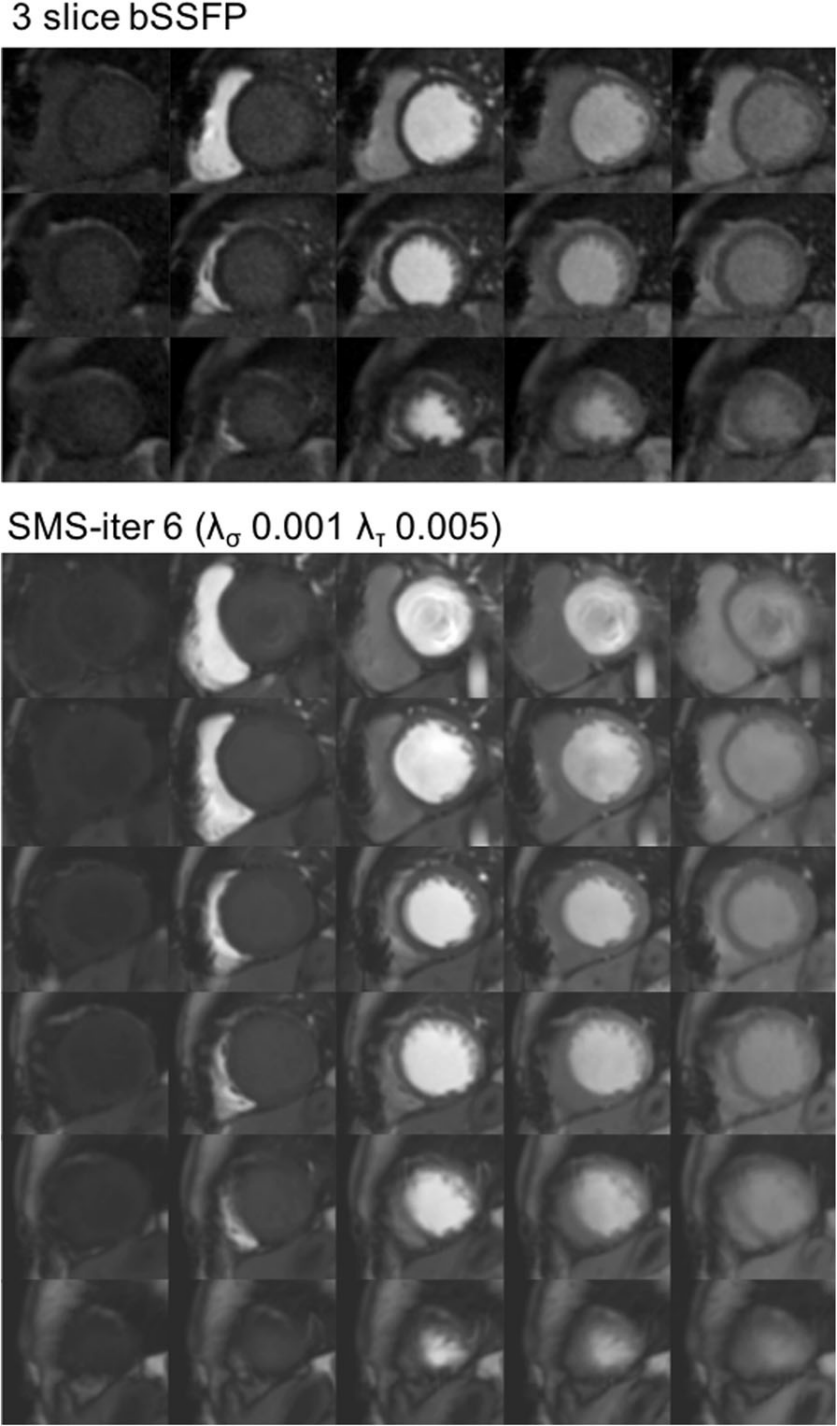
Dynamic perfusion series following contrast administration using 3 slice bSSFP and SMS-iter 6 (λ_σ_ 0.001 λ_τ_ 0.005) in patient 2. Top to bottom: base to apex. Left to right: baseline images, contrast transit through right ventricle, left ventricular blood pool, peak myocardial and washout. SMS-iter 6 (λ_σ_ 0.001 λ_τ_ 0.005) had better subjective image quality compared to 3 slice bSSFP


Overall, the optimal SMS iterative reconstruction method with the ranking for the best overall image quality (2.50 ± 0.53), least sequence related artifact (0.13 ± 0.35) and highest perceived SNR (2.25 ± 0.46) was SMS-iter 6 (λ_σ_ 0.001 λ_τ_ 0.005) (Table 3). This apparent difference is well illustrated in one patient as shown in Additional file 1: Video S1. Therefore, SMS-iter 6 (λ_σ_ 0.001 λ_τ_ 0.005) was chosen as the optimal SMS iterative reconstruction method for subsequent comparisons between 3 slice bSSFP and SMS-TGRAPPA. A comparison of 3 slice bSSFP and the optimum SMS iterative reconstruction method [SMS-iter 6 (λ_σ_ 0.001 λ_τ_ 0.005)] for two patients is presented in Figs. 1 and 2.

Additional file 1:**Video S1.** Dynamic perfusion images acquired from a mid-ventricular slice following contrast administration for a patient with a breath hold at peak contrast administration. The different reconstructions with simultaneous multi slice (SMS) with TGRAPPA and iterative reconstruction are presented. SMS-TGRAPPA was associated with poor SNR. SMS-iter 1 (λ_σ_ 0.0005 λ_τ_ 0.0005), with the least weighting for spatio-temporal regularisation had poor perceived SNR although moderate overall image quality. As the weighting of combined spatio-temporal regularisation increased, as with SMR-iter 4 (λ_σ_ 0.005 λ_τ_ 0.005), despite an improved SNR, there was greater sequence related artefact (particularly respiratory ghosting and image blurring) with a reduction in overall image quality. The optimum SMS reconstruction SMS-iter 6 (λ_σ_ 0.001 λ_τ_ 0.005) was determined to have the most favourable image quality, with the highest overall image quality, least sequence related artifacts and high perceived SNR. (MP4 5610 kb)


In two patients with suboptimal breath holds, there was degradation in overall image quality and increased sequence related artifact with increased respiratory ghosting in the iterative reconstruction parameters with greater spatial regularisation (λ_σ_) and temporal regularisation (λ_τ_), in particular for SMS-iter 4 (λ_σ_ 0.005 λ_τ_ 0.005). With SMS-iter 6 (λ_σ_ 0.001 λ_τ_ 0.005), the overall image quality remained diagnostic in these two patients, with good overall image quality and reduction in respiratory ghosting and high perceived SNR compared to SMS-iter 4 (λ_σ_ 0.005 λ_τ_ 0.005).

### Qualitative image assessment

**Fig. 3:**
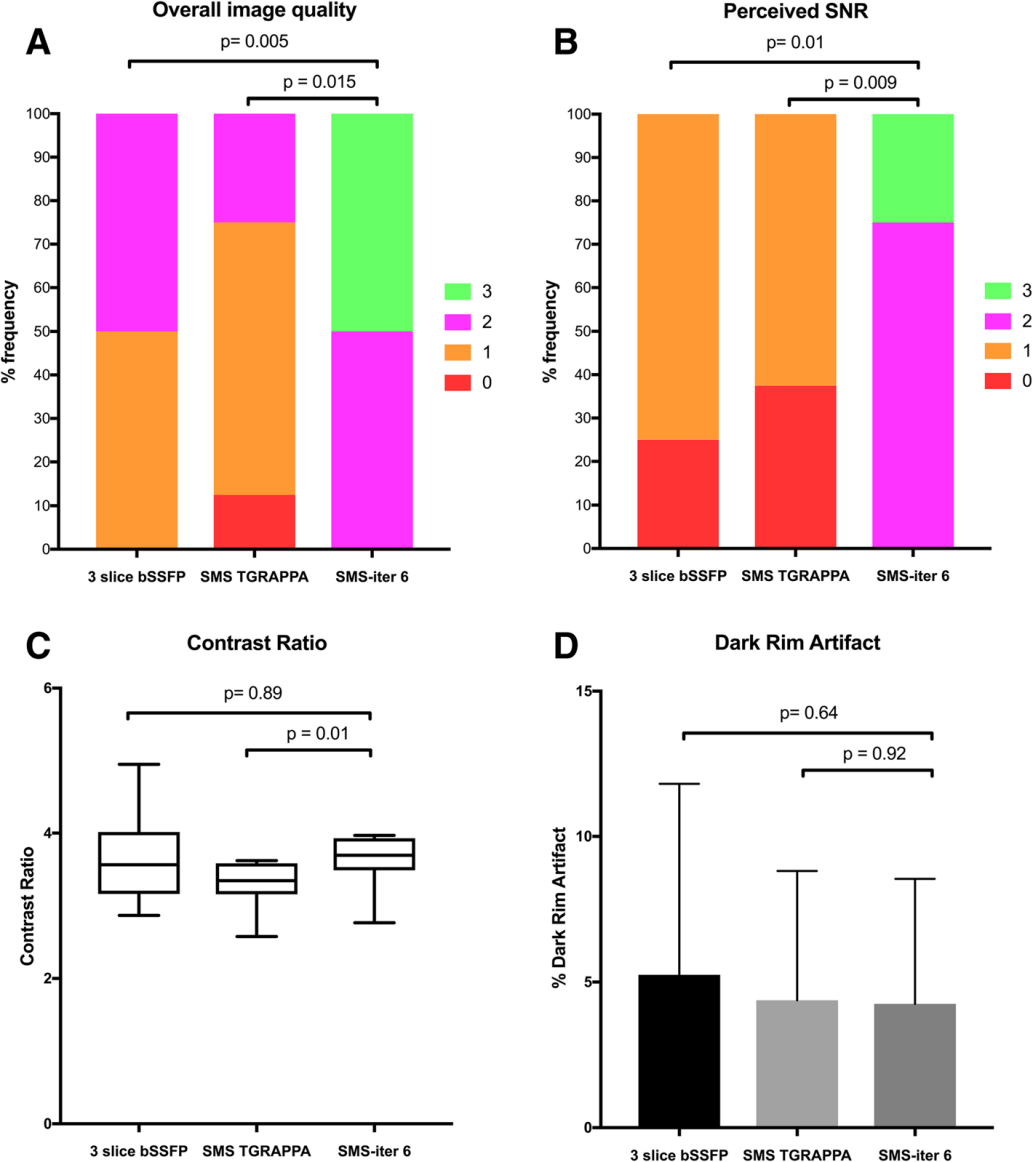
Comparison of 3 slice bSSFP, SMS-TGRAPPA and SMS-iter 6 (λ_σ_ 0.001 λ_τ_ 0.005) in 8 patients. **a** Overall diagnostic image quality. Scores for image quality range from 0 to 3 (0 = poor image quality and non-diagnostic, 1 = major artifact present but not limiting diagnosis, 2 = minor artifact present but not limiting diagnosis, 3 = excellent). Overall image quality was significantly higher with SMS-iter 6 (λ_σ_ 0.001 λ_τ_ 0.005) compared to SMS-TGRAPPA and 3 slice bSSFP. **b** Perceived Signal to Noise Ratio (SNR) with 3 slice bSSFP, SMS-TGRAPPA and SMS-iter 6 (λ_σ_ 0.001 λ_τ_ 0.005). Scores for perceived signal-to-noise (SNR) from 0 to 3 (0 = very poor SNR non-diagnostic image quality, 1 = major noise present but not limiting diagnosis, 2 = minor noise present but not limiting diagnosis, 3 = high SNR with excellent image quality). Perceived SNR was significantly higher with SMS-iter 6 (λ_σ_ 0.001 λ_τ_ 0.005) compared to SMS-TGRAPPA and 3 slice bSSFP. **c** Contrast ratio (CR; ratio peak blood pool signal intensity: peak myocardial signal intensity). There was no significant difference in the contrast ratio between SMS-iter 6 (λ_σ_ 0.001 λ_τ_ 0.005) and 3 slice bSSFP. **d** Dark rim artifact (mean and standard deviation): There was no significant difference in the percentage of dark rim artifact between 3 slice bSSFP and SMS-TGRAPPA or SMS-TGRAPPA and SMS-iter 6 (λ_σ_ 0.001 λ_τ_ 0.005)

SMS-iter 6 (λ_σ_ 0.001 λ_τ_ 0.005) had superior overall image quality (2.50 ± 0.53 vs 1.50 ± 0.53, *p* = 0.005) (Fig. 3a) and perceived SNR (2.25 ± 0.46 vs 0.75 ± 0.46, *p* = 0.010) compared to the 3 slice bSSFP (Fig. 3b). There was no significant difference in sequence related artifact with SMS-iter 6 (λ_σ_ 0.001 λ_τ_ 0.005) compared to the 3 slice bSSFP (0.13 ± 0.35 vs 2.50 ± 3.55, *p* = 0.14). Importantly, no banding artifact was observed in any of the SMS reconstructions over the myocardium.

With SMS-TGRAPPA compared to 3 slice bSSFP, there were no significant differences in overall image quality, sequence related artifact or perceived SNR.

SMS-iter 6 (λ_σ_ 0.001 λ_τ_ 0.005) compared to a SMS-TGRAPPA reconstruction had better overall image quality (2.50 ± 0.53 vs 1.13 ± 0.64, *p* = 0.015) and better perceived SNR (2.25 ± 0.46 vs 0.63 ± 0.52, *p* = 0.009). Interestingly, SMS-iter 6 (λ_σ_ 0.001 λ_τ_ 0.005) was associated with a reduction in sequence related artifacts compared to SMS-TGRAPPA (0.13 ± 0.35 vs 1.38 ± 1.79, *p* = 0.043) which was due to a reduction in respiratory ghosting.

Overall, there were no significant differences for patient related artifact between 3 slice bSSFP, SMS-TGRAPPA or SMS-iter 6 (λ_σ_ 0.001 λ_τ_ 0.005).

### Quantitative image assessment

SMS-iter 6 (λ_σ_ 0.001 λ_τ_ 0.005) had similar CR compared to 3 slice bSSFP (3.62 ± 0.39 vs 3.66 ± 0.65, *p* = 0.89) (Fig. 3c). There was no significant difference in CR between SMS-TGRAPPA and 3 slice bSSFP (3.30 ± 0.34 vs 3.66 ± 0.65, *p* = 0.20). CR with SMS-iter 6 (λ_σ_ 0.001 λ_τ_ 0.005) was higher than SMS-TGRAPPA (3.62 ± 0.39 vs 3.30 ± 0.34, *p* = 0.013). There were no significant differences in % DRA between 3 slice bSSFP and SMS-iter 6 (λ_σ_ 0.001 λ_τ_ 0.005) (5.25 ± 6.56 vs 4.25 ± 4.30, *p* = 0.64), 3 slice bSSFP and SMS-TGRAPPA (5.25 ± 6.56 vs 4.37 ± 4.43, *p* = 0.59) and SMS-TGRAPPA and SMS-iter 6 (λ_σ_ 0.001 λ_τ_ 0.005) (4.37 ± 4.43 vs 4.25 ± 4.30, *p* = 0.92) (Fig. 3d).

## Discussion

In this study we demonstrate the clinical feasibility of SMS contrast enhanced first-pass myocardial perfusion imaging with bSSFP, GC-LOLA, and iterative reconstruction at 1.5 T, which is a prerequisite prior to clinical evaluation in patients with suspected CAD for potential future clinical application. Doubled spatial coverage with preserved spatial resolution was achieved with SMS compared to a bSSFP approach. The employed iterative reconstruction technique of SMS data led to superior overall image quality, superior perceived SNR and similar CR compared to the 3 slice bSSFP. No banding artifacts were observed in any of the SMS perfusion images. Finally, a comprehensive image rating scale is proposed for application to development of myocardial perfusion sequences that may have utility to decipher optimal sequences and reconstruction methods.

Whole heart coverage for myocardial perfusion imaging is desirable as a strong correlation between CMR and nuclear perfusion studies has been demonstrated for the assessment of ischaemic burden [30], which is an important marker of prognosis [31]. High resolution myocardial perfusion imaging has been shown to improve detection of significant CAD through better detection of subendocardial ischaemia and less DRA [32]. In addition, high-resolution stress perfusion CMR allows for evaluation of transmural perfusion gradients to detect haemodynamically significant CAD [33, 34]. Currently, whole heart coverage can be achieved using 3D acquisition techniques which are associated with reduced in-plane spatial resolution while high resolution is achieved using multi-slice 2D acquisition protocols with limited spatial coverage (3–4 slices).

It is plausible that combining high resolution myocardial perfusion imaging with greater spatial coverage is advantageous for greater diagnostic accuracy and may provide a more accurate assessment of ischaemic burden. SMS may achieve the potential synergy of greater spatial coverage and high in-plane spatial resolution and this requires formal clinical evaluation in patients with CAD in a future clinical study.

The feasibility of CAIPIRINHA perfusion CMR has previously been demonstrated in a small cohort of healthy subjects using spoiled gradient recalled echo (GRE) readout [35–37] and combined with iterative regularised reconstruction with radial acquisition [38]. bSSFP pulse sequences for myocardial perfusion imaging are attractive due to better SNR and contrast-to-noise ratio (CNR) compared with GRE readout [39]. Previous work with CAIPIRINHA bSSFP myocardial perfusion imaging without GC-LOLA demonstrated an increased sensitivity of SMS to banding artifacts at 1.5 T [15]. In the present study, we combined CAIPIRINHA bSSFP with GC-LOLA and iterative reconstruction and observed no banding artifacts over the myocardium. The findings in the current study confirm previous work that GC-LOLA reduces SMS related banding artifacts [16]. Similar acquisition times were achieved compared to 3 slice bSSFP, which is important in clinical practice in order to avoid cardiac motion, particularly in stress perfusion imaging with greater heart rates.

Alternatively, SMS with bSSFP can be performed using blipped-CAIPI encoding where additional slice-gradient blips are employed to generate k-space phase modulation [40–42]. Although the employed approach has the potential to offer reduced sensitivity to eddy currents when compared to blipped-CAIPI encoding [16], future studies are warranted to compare both approaches.

There was lower perceived SNR for SMS-TGRAPPA compared to a 3 slice bSSFP approach. We consider the reduction in perceived SNR for SMS-TGRAPPA to be related to additional g-factor noise amplification [13, 43], which increases with the overall acceleration factor. However, the potential loss in SNR was recovered with the optimal iterative reconstruction parameters and resulted in an improved overall image quality and perceived SNR compared to a bSSFP approach and SMS-TGRAPPA.

Sequence related artifacts increased with greater spatial and temporal regularisation and reduced overall image quality. There was also a trend for increased respiratory ghosting in iterative reconstruction of SMS data with greater spatial regularisation (λ_σ_) which indicates that such reconstruction parameters may not be suitable in patients with poor breath holds. In addition, in two patients with poor breath holds, increased artifacts were observed with a reduction in image quality in reconstructions with high spatial and temporal regularisation. However, using the optimal iterative reconstruction, there was a reduction in sequence related artifact and diagnostic image quality was achieved in all patients including two patients with poor breath holding.

Using the rankings obtained from the rating scheme presented in Table 1, SMS-iter 6 (λ_σ_ 0.001 λ_τ_ 0.005) was identified as the optimal imaging reconstruction parameters for SMS imaging, selected by the best overall image quality and perceived SNR and lowest frequency of sequence related artifact. Using this detailed rating scheme allowed us to carefully decipher the optimal reconstruction parameters for the range of iterative reconstruction from 56 imaging datasets.

**Fig. 4:**
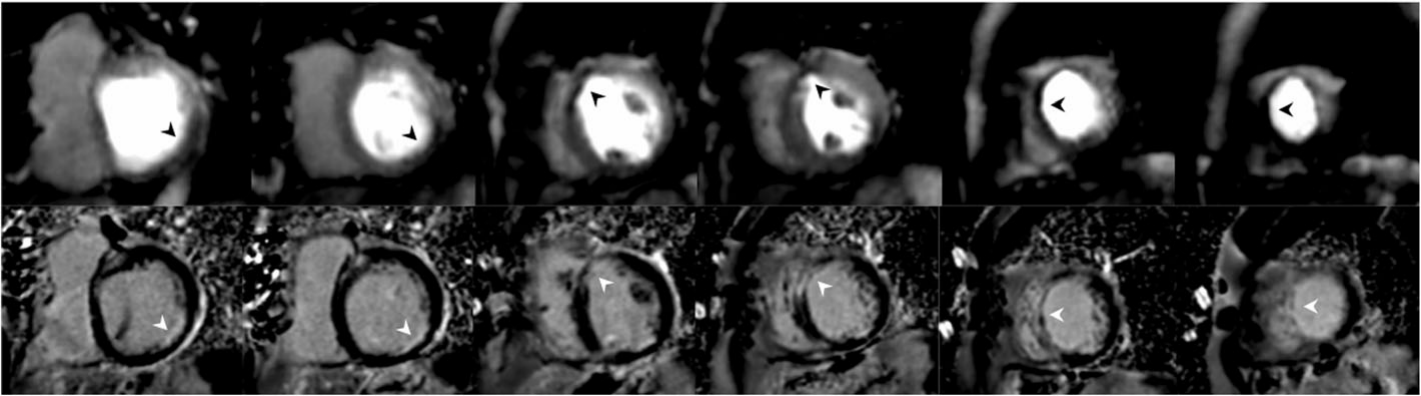
Peak myocardial perfusion signal intensity images and late gadolinium enhancement (LGE) images of a patient with subendocardial myocardial infarction. Top panel: peak myocardial dynamic frame for SMS-iter 6 (λ_σ_ 0.001 λ_τ_ 0.005); bottom panel: LGE imaging following contrast administration. The rest perfusion defects (black arrows) matched with the areas of subendocardial scar (white arrows)

In this study, we undertook myocardial perfusion imaging at rest without the administration of intravenous vasodilatory stress agents. In one patient with an ischaemic cardiomyopathy, and subendocardial myocardial infarction, rest perfusion imaging correctly delineated perfusion defects with areas of subendocardial scar on late gadolinium enhancement imaging (Fig. 4). This indicates a signal for potential utility for application for ischaemia testing with SMS.

Vasodilatory stress increases myocardial blood flow (MBF) up to five-fold in healthy individuals, and in turn leads to a significant increase in signal intensity. This magnitude of signal intensity increase is not seen with rest perfusion imaging and this may reflect the overall lower global image quality observed in the 3 slice bSSFP and SMS TGRAPPA. Nevertheless, by using rest perfusion imaging alone, with a lower resting MBF and subsequent lower signal intensity, the standards and benchmark for comparison are higher.

There are various confounding physiological factors when comparing repeated stress perfusion imaging due to absolute changes in haemodynamic responses [44], MBF, signal intensity and therefore image quality. Hence, for the purpose of this study, which serves to demonstrate the feasibility of combining SMS, bSSFP, GC-LOLA and iterative reconstruction, rest perfusion imaging only was used. Therefore, this study serves as an important step for the methods development prior to a clinical validation study in patients with stress perfusion imaging.

The prolonged computation times for iterative reconstruction for SMS data may pose a barrier to implementation into routine clinical practice. While iterative reconstruction can significantly improve CMR image quality, such an approach is computationally intensive compared to standard reconstruction [45]. Techniques to reduce reconstruction time such as by use of a graphics processing unit are feasible, have been applied to CMR data with iterative reconstruction [46], with substantial increase of reconstruction speed [47]. Hence, rapid iterative reconstruction of SMS data may be feasible with dedicated hardware and thereby facilitate implementation into routine clinical care. 

### Future work

A slice acceleration factor of 2 was used for this study which resulted in acquisition of 6 slices. For true whole heart coverage with contiguous slice coverage and in particular the true apical cap, a slice acceleration factor of 3 or 4 would be required, but this would require a trade off against any potential g-factor noise amplification.

Myocardial perfusion imaging at 3 Tesla is highly desirable with benefit of increased SNR and contrast-to-noise (CNR), which can be traded off with higher acceleration with parallel imaging which comes with an SNR penalty [48]. Hence, SMS bSSFP GC-LOLA with iterative reconstruction could be evaluated at 3 T field strength and is well suited for greater slice acceleration. However, increasing field strength may also lead to an increase in field inhomogeneities and in turn lead to greater banding artifact. Careful shimming and selection of the optimal frequency from a frequency scout can be used to minimise off-resonance artifacts [49].

In this study we demonstrated the feasibility of SMS in patients with rest perfusion imaging only in order to ascertain diagnostic image quality and determine the optimal reconstruction parameters for SMS imaging as a methods development study. In order to validate the clinical application of this technique, vasodilator stress in a large cohort of patients with suspected CAD would be required. This larger cohort would need to reflect the wide distribution of disease of CAD (single vessel, two vessel and multivessel) in addition to the variation of clinical factors (arrhythmias, poor breath holders and obese patients) encountered in clinical practice. This current study now paves the way for such a clinical study in a group of patients with correlation of ischaemia related perfusion with invasive coronary angiographic fractional flow reserve data and/or positron emission tomography (PET).

### Limitations

The sample size for this study is quite modest, but the purpose of this study was to determine the feasibility of SMS bSSFP with GC-LOLA and iterative reconstruction and to compare to a standard sequence used for routine clinical practice.

Undertaking SNR and CNR measurements with parallel imaging are challenging and the added complexity in this study is that iterative reconstruction inherently thresholds and shrinks noise inhomogenously across the field of view [50]. Studies with compressed sensing have avoided reporting absolute SNR measurements [51] and reported visual perception of SNR on a four point scale [50]. In this study, we also reported perceived SNR with detailed explanation of each parameter and calibration between observers. In addition, we reported CR to provides a metric for quantitative image quality, which is not absolute SNR or CNR, but provides a meaningful ratio for image quality from dynamic perfusion images.

A minimum duration of 10 min is recommended between repeat contrast myocardial perfusion imaging [52], although contrast retention is often observed with longer periods. This study protocol used a minimum washout period of 15 min between each contrast administration. The time period for washout of contrast may have influenced the baseline signal intensity for the second rest perfusion study undertaken, although by alternating the order of sequences in each successive patient, we attempted to counterbalance the overall effect in this cohort of patients.

While 7 different combinations of weighting for spatial and temporal regularisation were employed, further combinations could have explored the effect of greater spatial and / or temporal regularisation. However, by using a step wise range of permutations for regularisation, we attempted to encompass a wide range of possible reconstructions parameters. In addition, greater regularisation may artificially over-smooth the images, with loss of important spatial and temporal data for dynamic perfusion imaging and hence we chose to limit the extent of regularisation.

The number of slices for comparison between 3 slice bSSFP and 6-slice SMS were not equal, and this may have influenced the comparability of the ratings presented in Table 1. In any case, such an effect would bias against the new proposed technique of SMS as if an artifact was observed in 1/3 bSSFP images, this would score the same as an artifact in 1 or 2 SMS slices. Image ratings were performed with all slices together for each perfusion sequence rather than single slices in isolation in order to allow global assessment of image quality and artifacts. Ratings could have been taken individually for each slice, although in clinical practice, dynamic perfusion images are interpreted collectively rather than on an individual slice, and hence we chose to rate all perfusion slices for each dataset collectively.

## Conclusion

Contrast enhanced myocardial perfusion imaging using SMS bSSFP with GC-LOLA and iterative reconstruction is feasible and provides improved image quality, doubled spatial coverage and identical in-plane spatial resolution compared to a 3 slice bSSFP approach. This technique may represent a route to achieve high resolution 3D whole heart coverage for improved diagnostic accuracy, identification of subendocardial ischaemia and assessment of ischaemic burden in patients with suspected CAD. A clinical validation study in patients with CAD is now warranted.

## Abbreviations

bSSFP: balanced Steady State Free Precession
CAD: Coronary Artery Disease
CAIPIRINHA: Controlled Aliasing in Parallel Imaging Results in Higher Acceleration
CMR: Cardiovascular Magnetic Resonance
CNR: Contrast-to-Noise Ratio
CR: Contrast ratio
DRA: Dark Rim Artifact
ECG: Electrocardiogram
EDV: End Diastolic Volume
FISTA: Fast Iterative Shrinkage Thresholding Algorithm
FOV: Field of view
GC-LOLA: Gradient Controlled Local Larmor Adjustment
GRAPPA: Generalized Autocalibrating Partial Parallel Acquisition
LV: Left Ventricle/Left ventricular
LVEF: Left ventricular ejection fraction
LVOT: Left Ventricular Outflow Tract
MBF: Myocardial blood flow
PET: Positron Emission Tomography
POMP: Phase Offset Multiplanar
RF: Radiofrequency
ROI: Region-of-interest
RVEF: Right ventricular ejection fraction
SENSE: Sensitivity Encoding
SI: Signal Intensity
SMS: Simultaneous Multi Slice
SMS-iter: Simultaneous Multi Slice with Iterative reconstruction
SNR: Signal to Noise Ratio
TE: echo time
TGRAPPA: Temporal GRAPPA
TI: inversion time
TR: repetition time
